# Twist promotes reprogramming of glucose metabolism in breast cancer cells through PI3K/AKT and p53 signaling pathways

**DOI:** 10.18632/oncotarget.4697

**Published:** 2015-07-27

**Authors:** Li Yang, Yixuan Hou, Jie Yuan, Shifu Tang, Hailong Zhang, Qing Zhu, Yan-e Du, Mingli Zhou, Siyang Wen, Liyun Xu, Xi Tang, Xiaojiang Cui, Manran Liu

**Affiliations:** ^1^ Key Laboratory of Laboratory Medical Diagnostics, Chinese Ministry of Education, Chongqing Medical University, Chongqing 400016, China; ^2^ Experimental Teaching Center of Basic Medicine Science, Chongqing Medical University, Chongqing 400016, China; ^3^ Department of Endocrine and Breast Surgery, The First Affiliated Hospital of Chongqing Medical University, Chongqing 400016, China; ^4^ Department of Surgery, Department of Obstetrics and Gynecology, Samuel Oschin Comprehensive Cancer Institute, Cedars-Sinai Medical Center, Los Angeles, CA 90048, USA

**Keywords:** glucose metabolism, Twist, PI3K/AKT, p53

## Abstract

Twist, a key regulator of epithelial-mesenchymal transition (EMT), plays an important role in the development of a tumorigenic phenotype. Energy metabolism reprogramming (EMR), a newly discovered hallmark of cancer cells, potentiates cancer cell proliferation, survival, and invasion. Currently little is known about the effects of Twist on tumor EMR. In this study, we found that glucose consumption and lactate production were increased and mitochondrial mass was decreased in Twist-overexpressing MCF10A mammary epithelial cells compared with vector-expressing MCF10A cells. Moreover, these Twist-induced phenotypic changes were augmented by hypoxia. The expression of some glucose metabolism-related genes such as PKM2, LDHA, and G6PD was also found to be upregulated. Mechanistically, activated β1-integrin/FAK/PI3K/AKT/mTOR and suppressed P53 signaling were responsible for the observed EMR. Knockdown of Twist reversed the effects of Twist on EMR in Twist-overexpressing MCF10A cells and Twist-positive breast cancer cells. Furthermore, blockage of the β1-integrin/FAK/PI3K/AKT/mTOR pathway by siRNA or specific chemical inhibitors, or rescue of p53 activation can partially reverse the switch of glucose metabolism and inhibit the migration of Twist-overexpressing MCF10A cells and Twist-positive breast cancer cells. Thus, our data suggest that Twist promotes reprogramming of glucose metabolism in MCF10A-Twist cells and Twist-positive breast cancer cells via activation of the β1-integrin/FAK/PI3K/AKT/mTOR pathway and inhibition of the p53 pathway. Our study provides new insight into EMR.

## INTRODUCTION

Most cancer cells tend to utilize aerobic glycolysis to produce energy instead of oxidative phosphorylation even in the presence of abundant oxygen. This phenomenon was first described by Otto Warburg and termed as Warburg effect [1]. Subsequently Warburg effect has been proven to exist widely in various tumors. Altered energy metabolism is now defined as a hallmark of cancer [2–4]. The metabolic switch, or energy metabolism reprogramming (EMR), has attracted much attention recently, as evidenced by many reviews on this topic [5–7]. Hexokinase 2 (HK2), pyruvate kinase 2 (PKM2) and lactate dehydrogenase A (LDHA) were reported to regulate EMR. HK2 catalyzes the essentially irreversible first step of glycolysis by phosphorylating glucose to glucose-6-phosphate. PKM2 is the key regulator at the rate-limiting final step of glycolysis by converting phosphoenolpyruvic acid into pyruvate acid. LDHA catalyzes the interconversion of pyruvate and lactate. It has been shown that abnormal expression of these enzymes could contribute to promote Warburg effect in cancer cells [8–11]. Cancer cells reprogram their metabolism to facilitate fast proliferation through increased glycolysis and biosynthetic activities. EMR is essential to the survival of cancer cells and can increase the proliferation, migration and invasion of cancer cells [12–15]. To date, the key molecular mechanisms for EMR regulation and its role in cancer development still remain elusive.

Twist, a highly conserved basic Helix-Loop-Helix transcription factor, is involved in the regulation of various physiological and pathological processes such as organ development, cell proliferation, differentiation, and tumorigenesis. Twist is known to function as a major regulator in EMT and thereby promotes tumor invasion and metastasis [16–18]. Studies have increasingly demonstrated that Twist plays an important role in tumor development. Nevertheless, it still remains unclear whether Twist regulates EMR.

Our cDNA microarray and proteomics analysis indicated that the expression of some glucose metabolism-related genes in Twist-overexpressing MCF10A human mammary epithelial cells (defined here MCF10A-Twist) was changed (data unpublished). We have reported that Twist induces EMT and promotes migration and invasion in MCF10A-Twist [19]. In this study, the effects of Twist on EMR of MCF10A-Twist were explored. We detected EMR-related phenotypes such as changes in glucose consumption, lactate production, and mitochondrial mass in MCF10A-Twist compared with MCF10A-Vector under normoxic and hypoxic conditions, respectively. Our results demonstrate that Twist can induce EMR in MCF10A-Twist cells. The β1-integrin/FAK/PI3K/AKT/mTOR and p53 signaling pathways are important mediators for the process. These data may advance our understanding of tumor development and provide new strategies for prevention and treatment of breast cancer.

## RESULTS

### Twist invokes the altered energy metabolic phenotype in MCF10A-Twist cells

**Figure 1:**
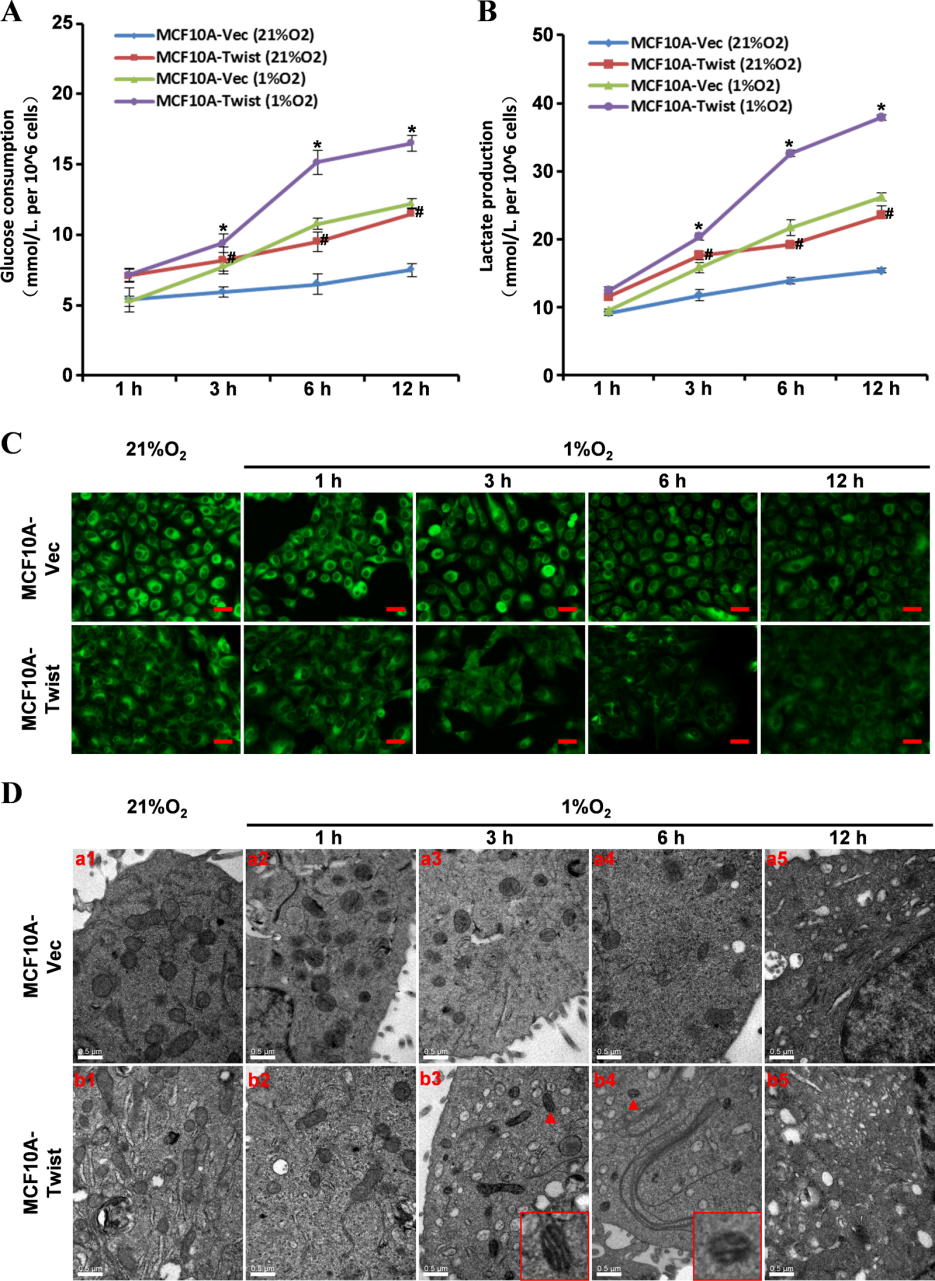
The altered energy metabolic phenotype in Twist-overexpressing MCF10A cells **A, B.** Glucose consumption and lactate production were measured in MCF10A-Vector and MCF10A-Twist cells (MCF10A-Twist cells versus MCF10A-Vector cells. #*P* < 0.05, under normal oxygen condition; *P < 0.05, under hypoxia condition). **C.** Fluorescence microscope analysis of mitochondrial mass in MCF10A-Vector and MCF10A-Twist cells after Mito-Tracker Green staining (Magnification, x200. Scale bars, 100 μm). **D.** Mitochondrial morphological analysis in MCF10A-Vector and MCF10A-Twist cells by transmission electron microscope (Magnification, x25000. Scale bars, 0.5 μm).

After MCF10A-Vector and MCF10A-Twist cells were incubated with 21% O_2_ or 1% O_2_ for 1, 3, 6 and 12 h, the effect of Twist on glucose consumption was investigated. The increased glucose consumption was detected by using Glucose Assay Kit in MCF10A-Twist cells compared with MCF10A-Vector cells under normoxic (21% O_2_) or hypoxic (1% O_2_) conditions. Hypoxic treatment could increase glucose consumption of MCF10A-Twist cells compared with MCF10A-Vector cells (Fig. 1A).

To examine whether the glycolysis was altered by Twist, lactate production was detected using Lactate Assay Kit. As shown in Fig. 1B, MCF10A-Twist cells produced more lactate than MCF10A-Vector cells under normoxic or hypoxic conditions. Hypoxic treatment further increased lactate generation in MCF10A-Twist cells compared with MCF10A-Vector cells.

Mito-Tracker Green, a fluorescent probe of mitochondria, was used to study the effect of Twist on mitochondrial mass in MCF10A cells. Compared with MCF10A-Vector cells, MCF10A-Twist cells presented weaker fluorescence intensity, suggesting these cells had lower mitochondrial mass than control cells. Moreover, mitochondrial mass of MCF10A-Twist was further reduced under hypoxic conditions (Fig. 1C) in contrast to MCF10A-Vector cells.

To further investigate mitochondrial function, the number and morphology of mitochondria were observed by transmission electron microscopy (TEM). There were fewer mitochondria observed in the MCF10A-Twist cells (Fig. 1D, b1) compared with that in MCF10A-Vector cells (Fig. 1D, a1) under normoxic conditions. The number of mitochondria in both MCF10A-Vector and -Twist cells was gradually reduced with the increasing hypoxic exposure time, and less mitochondria were in MCF10A-Twist cells (Fig. 1D, b2–b5) than in MCF10A-Vector cells (Fig. 1D, a2–a5). Moreover, the longitudinal mitochondrial crest (Fig. 1D, b3) and swollen mitochondria (Fig. 1D, b4) could be seen in MCF10A-Twist but not in control cells after hypoxia exposure.

### Loss of Twist expression partly reverses the switch of energy metabolism

**Figure 2:**
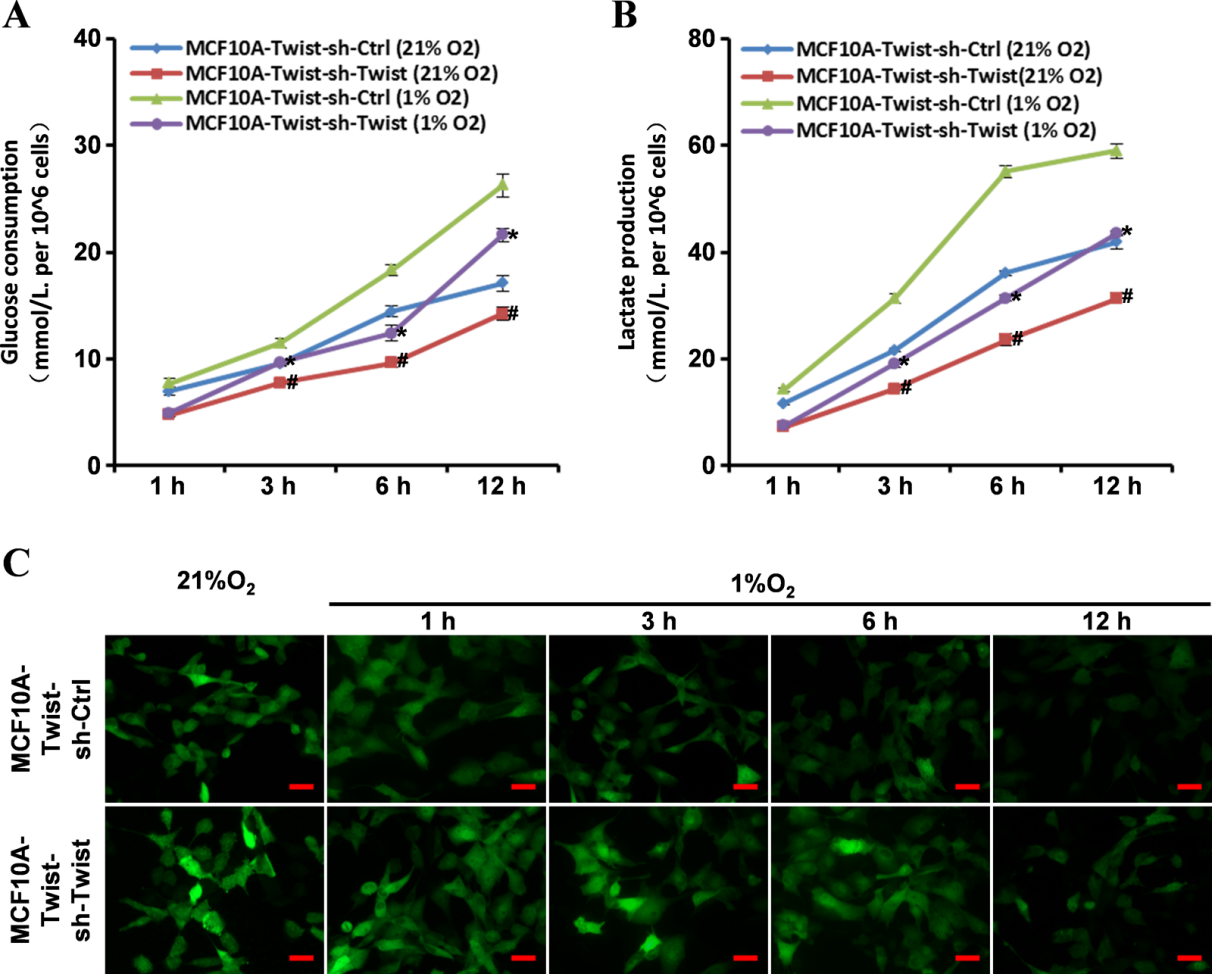
Loss of Twist expression reverses the altered energy metabolic phenotype in MCF10A-Twist cells **A, B.** Glucose consumption and lactate production were measured in MCF10A-Twist-sh-Ctrl and MCF10A-Twist-sh-Twist cells (MCF10A-Twist-sh-Twist cells versus MCF10A-Twist-sh-Ctrl cells. #*P* < 0.05, under normal oxygen condition; *P < 0.05, under hypoxia condition). **C.** Fluorescence microscope analysis of mitochondrial mass in MCF10A-Twist-sh-Ctrl and MCF10A-Twist-sh-Twist cells after Mito-Tracker Green staining (Magnification, x200. Scale bars, 100 μm).

To further study the role of Twist in regulating EMR, we tested whether Twist silence in MCF10A-Twist and Twist-positive breast cancer cells could reverse the energy metabolic phenotype. Using a lentivirus vector expressing human Twist shRNA, Twist-silenced MCF10A-Twist (MCF10A-Twist-sh-Twist) and BT549 (BT549-sh-Twist) cells were successfully established (Supplemental Fig. 1A–1D). Knockdown of Twist in MCF10A-Twist (MCF10A-Twist-sh-Twist) decreased glucose consumption and lactate production compared with control cells (MCF10A-Twist-sh-Ctrl) (Fig. 2A–2B). Hypoxic exposure rendered MCF10A-Twist cells (MCF10A-Twist-sh-Ctrl) to consume more glucose and produce more lactate than Twist-silenced MCF10A-Twist cells (MCF10A-Twist-sh-Twist) (Fig. 2A–2B). This was further confirmed in BT549-sh-Twist cells (Supplemental Fig. 1E–1F). The mitochondrial mass was partly increased in MCF10A-Twist-sh-Twist and BT549-sh-Twist (Fig. 2C and Supplemental Fig. 1G).

### Expression of energy metabolism-associated genes is regulated by Twist in MCF10A-Twist and Twist-positive breast cancer cells

**Figure 3:**
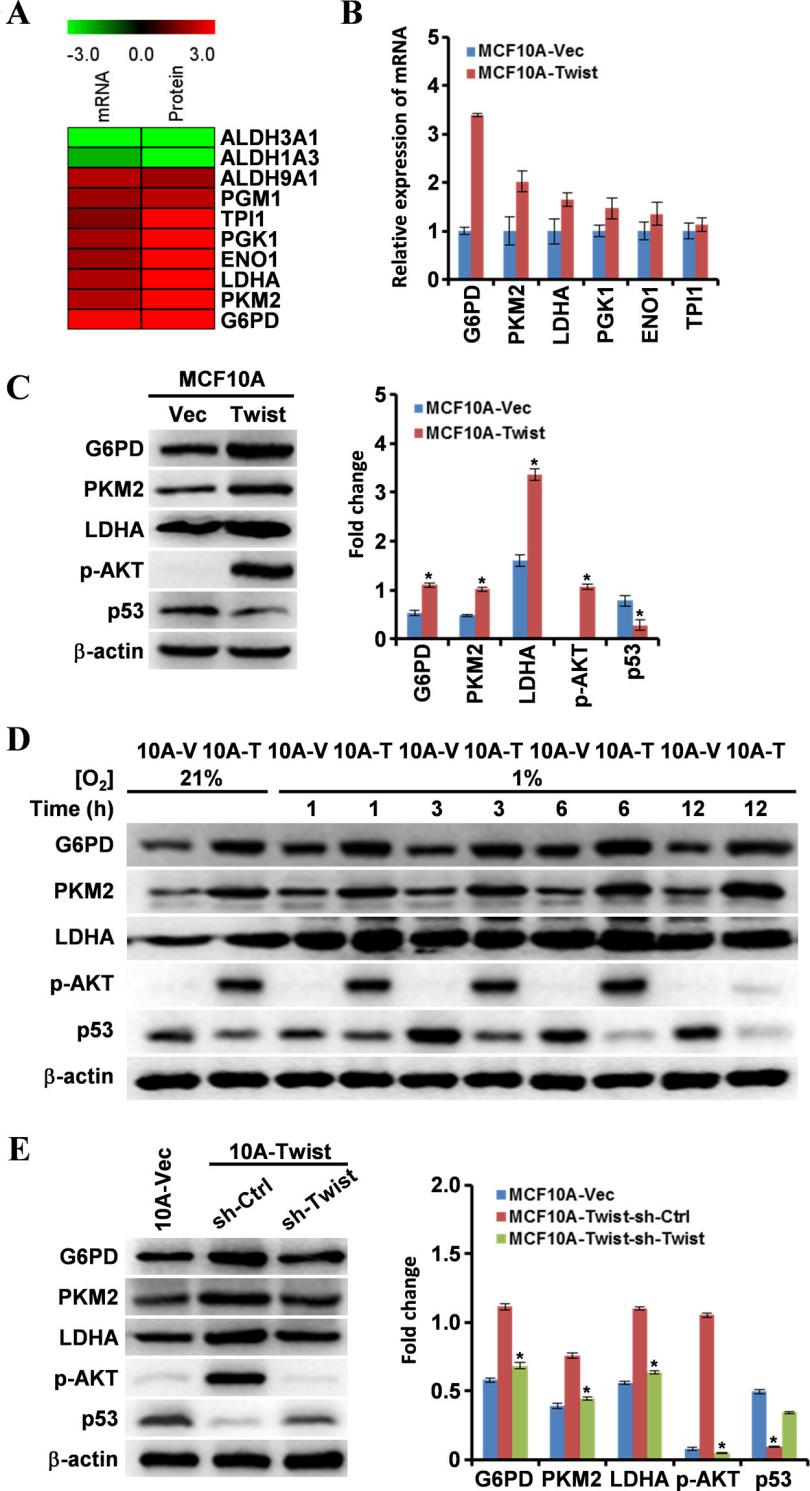
Expression of genes associated with cell energy metabolism is altered in MCF10A-Twist cells **A.** The differential expression genes related to cell energy metabolism were identified after bioinformatics analysis of our mRNA microarray data and proteomic data of MCF10A-Twist and MCF10A-Vector cells. **B.** The mRNA levels of G6PD, PKM2, LDHA, PGK1, ENO1, and TPI1 were analyzed by qRT-PCR in MCF10A-Vector and MCF10A-Twist cells. **C, D, E.** Western blotting analysis was used to determine the expression of G6PD, PKM2, LDHA, p-AKT and p53. β-actin was used as an internal control. (C) Protein levels in MCF10A-Vector and MCF10A-Twist cells. (*P < 0.05). (D) Protein levels in MCF10A-Vector and MCF10A-Twist cells after hypoxia treatment for indicated time. (E) Protein levels in MCF10A-Twist-sh-Ctrl and MCF10A-Twist-sh-Twist cells. (*P < 0.05).

To understand the molecular mechanism of Twist-driven EMR, we analyzed our cDNA microarray and proteomic data of MCF10A-Twist and MCF10A-Vector cells. Indeed, a set of energy metabolism-associated genes were dysregulated in MCF10A-Twist compared with MCF10A-Vector (Fig. 3A). Some of these genes were validated using qRT-PCR analysis. It was found that G6PD, PKM2, LDHA, PGK1, ENO1 and TPI1 were up-regulated in MCF10A-Twist (Fig. 3B). Expression of PKM2, LDHA and G6PD, which are critical genes linked to energy metabolism, was further confirmed by western blotting (Fig. 3C). Moreover, the level of p-AKT was up-regulated, while p53 was down-regulated in MCF10A-Twist (Fig. 3C). These two genes were reported to be related with energy metabolic switch [20, 21]. Levels of p-AKT and p53 in Twist-positive BT549 cells were higher than those in MCF10A cells (Supplemental Fig. 2A). Interestingly, levels of G6PD, PKM2, LDHA and p-AKT in MCF10A-Twist (Fig. 3D) and BT549 (Supplemental Fig. 2B) were increased under hypoxic conditions. In addition, Twist knockdown led to decreased G6PD, PKM2, LDHA, p-AKT and elevated p53 in MCF10A-Twist-sh-Twist and BT549-sh-Twist as expected (Fig. 3E and Supplemental Fig. 2C). These findings indicate that PI3K/AKT and p53 signaling may be involved in the Twist-driven EMR.

### Twist induces EMR by activation of PI3K/AKT/mTOR signaling and inhibition of p53 signaling

**Figure 4:**
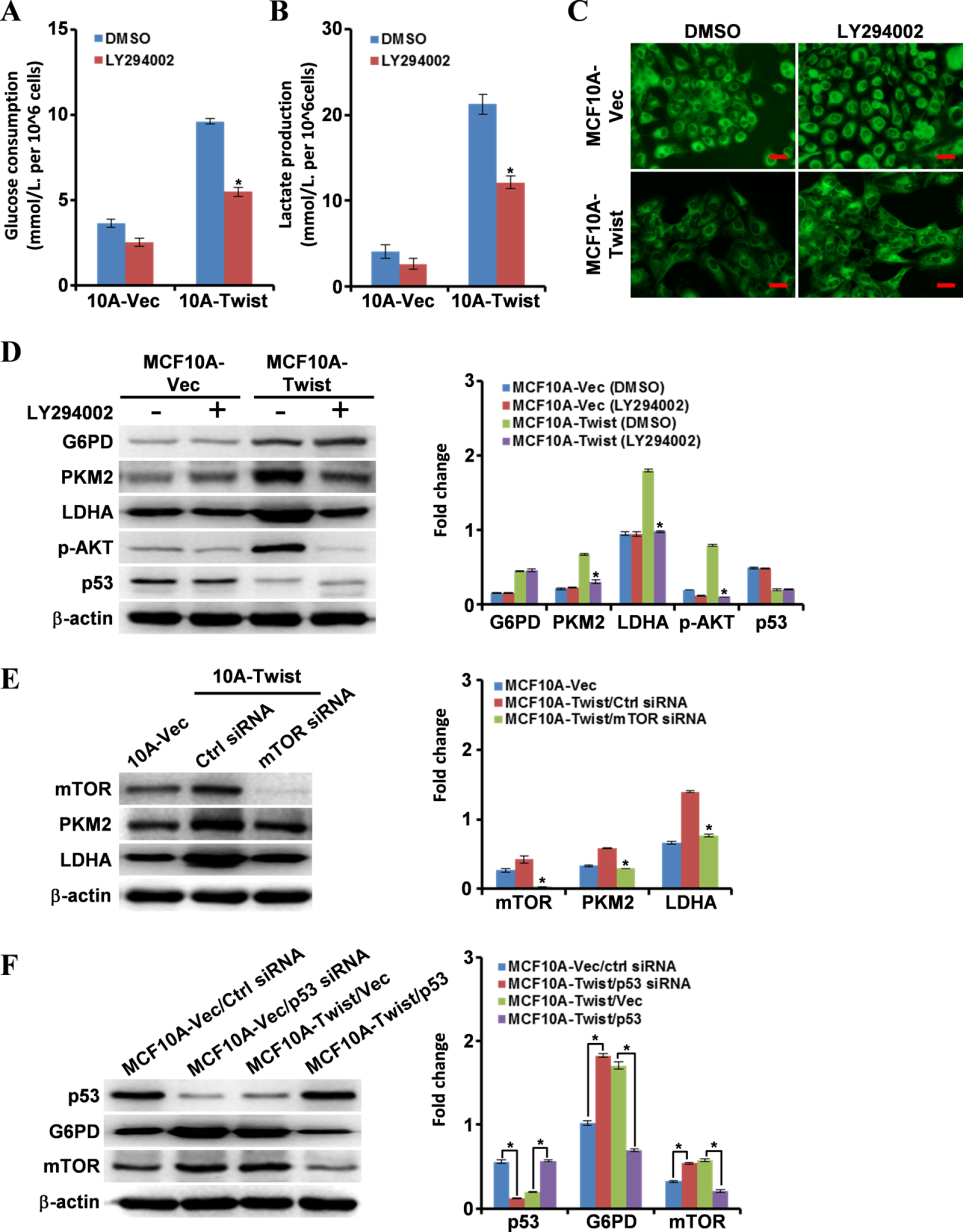
Molecular mechanisms underlying cell energy metabolism reprogramming in MCF10A-Twist cells **A, B.** After treatment with LY294002, glucose consumption and lactate production of MCF10A-Vector and MCF10A-Twist cells were detected. (*P < 0.05). **C.** Fluorescence microscope analysis of mitochondrial mass in MCF10A-Vector and MCF10A-Twist cells treated with LY294002 by Mito-Tracker Green staining (Magnification, x200. Scale bars, 100 μm). **D.** After treatment with LY294002, expression of G6PD, PKM2, LDHA, p-AKT, and p53 in MCF10A-Vector and MCF10A-Twist cells was determined by Western blotting. β-actin was used as an internal control (*P < 0.05). **E.** Western blotting was used to analyze the mTOR, PKM2 and LDHA expression in MCF10A-Vector, MCF10A-Twist transfected with control siRNA, MCF10A-Twist transfected with mTOR siRNA (*P < 0.05). β-actin was used as an internal control. **F.** Western blotting was applied to analyze the p53, mTOR and G6PD levels in the indicated cells (*P < 0.05). β-actin was used as an internal control.

To explore the role of PI3K/AKT and p53 in Twist-induced EMR, the PI3K inhibitor, LY294002, was used to treat MCF10A-Twist and BT549 cells. As expected, glucose consumption and lactate production in MCF10A-Twist (Fig. 4A–4B) and BT549 cells (Supplemental Fig. 3A–3B) were decreased, whereas mitochondrial mass was increased (Fig. 4C and Supplemental Fig. 3C).

In addition, expression of PKM2 and LDHA in MCF10A-Twist and BT549 cells was down-regulated by LY294002 (Fig. 4D and Supplemental Fig. 3D). Of note, there were no changes of p53 and G6PD expression after LY294002 treatment. PKM2 has been identified to be a target gene of mTOR [22], a downstream effector in the PI3K/AKT pathway [23, 24]. Silencing mTOR with siRNA decreased PKM2 and LDHA protein levels in MCF10A-Twist and BT549 (Fig. 4E and Supplemental Fig. 3E), suggesting that PI3K/AKT/mTOR regulates PKM2 and LDHA expression in MCF10A-Twist and BT549. Moreover, glucose consumption and lactate production in MCF10A-Twist/mTOR siRNA (Supplemental Fig. 4A–4B) and BT549/mTOR siRNA (Supplemental Fig. 4C–4D) were also decreased compared to their control cells.

It was demonstrated that loss of p53 function might activate the pentose phosphate pathway (PPP) and contribute to Warburg effect [25]. Indeed, p53 (which is wild type in MCF10A [26]) was down-regulated, whereas the key rate-limiting enzyme of PPP G6PD was upregulated, in MCF10A-Twist (Fig. 3C). To confirm the negative correlation between p53 and G6PD in MCF10A-Twist, the wild-type p53 gene was transfected into MCF10A-Twist. As expected, restoration of p53 expression in MCF10A-Twist suppressed G6PD expression (Fig. 4F). To extend this finding, the endogenous p53 and G6PD in the representative breast cancer cell line MCF-7 (Twist-low level) and BT549 (Twist-high level) were detected. Low levels of wild-type p53 [27] and high levels of G6PD were detected in MCF-7, and high levels of mutant p53 [28] and low levels of G6PD were identified in BT549. However, mild levels of p53 and G6PD were in MCF10A (Supplemental Fig. 3F). Unlike wild-type p53, the p53 mutants (e.g. R175H, R273H, and G279E) were shown to possess minimal or no activity in inhibiting G6PD [25]. Therefore, there were no obvious changes in the level of G6PD expression in BT549 after knockdown of p53 with siRNA (Supplemental Fig. 3G). To further investigate the role of p53 in modulating G6PD, the wild-type p53 was stably transfected into BT549. Consistent with our expectation, G6PD expression was partly repressed by wild-type p53 in BT549/p53 (Supplemental Fig. 3H). Our data indicate that Twist might increase G6PD by repressing wild-type p53, thus promoting PPP.

Moreover, it has been indicated that the mTOR pathway is negatively regulated by the p53 tumor suppressor [29, 30]. Consistent with this, our data show that restoration of wild-type p53 expression in MCF10A-Twist (Fig. 4F) and Twist-positive BT549 cells (Supplemental Fig. 3H) inhibited mTOR expression. Subsequently, the EMR biomarker of glucose consumption and lactate production in MCF10A-Twist/p53 and BT549/p53 were determined. As expected, glucose consumption and lactate production in MCF10A-Twist/p53 (Supplemental Fig. 5A–5B) and BT549/p53 (Supplemental Fig. 5C–5D) were decreased. These data suggest that wild-type p53 exerts negative regulation of mTOR pathway, however, Twist relieves this inhibition by repressing wild-type p53, and thus promotes glycolysis.

### β1-integrin/FAK signaling is responsible for the activation of PI3K/AKT/mTOR signaling axis and thus promotes EMR

**Figure 5:**
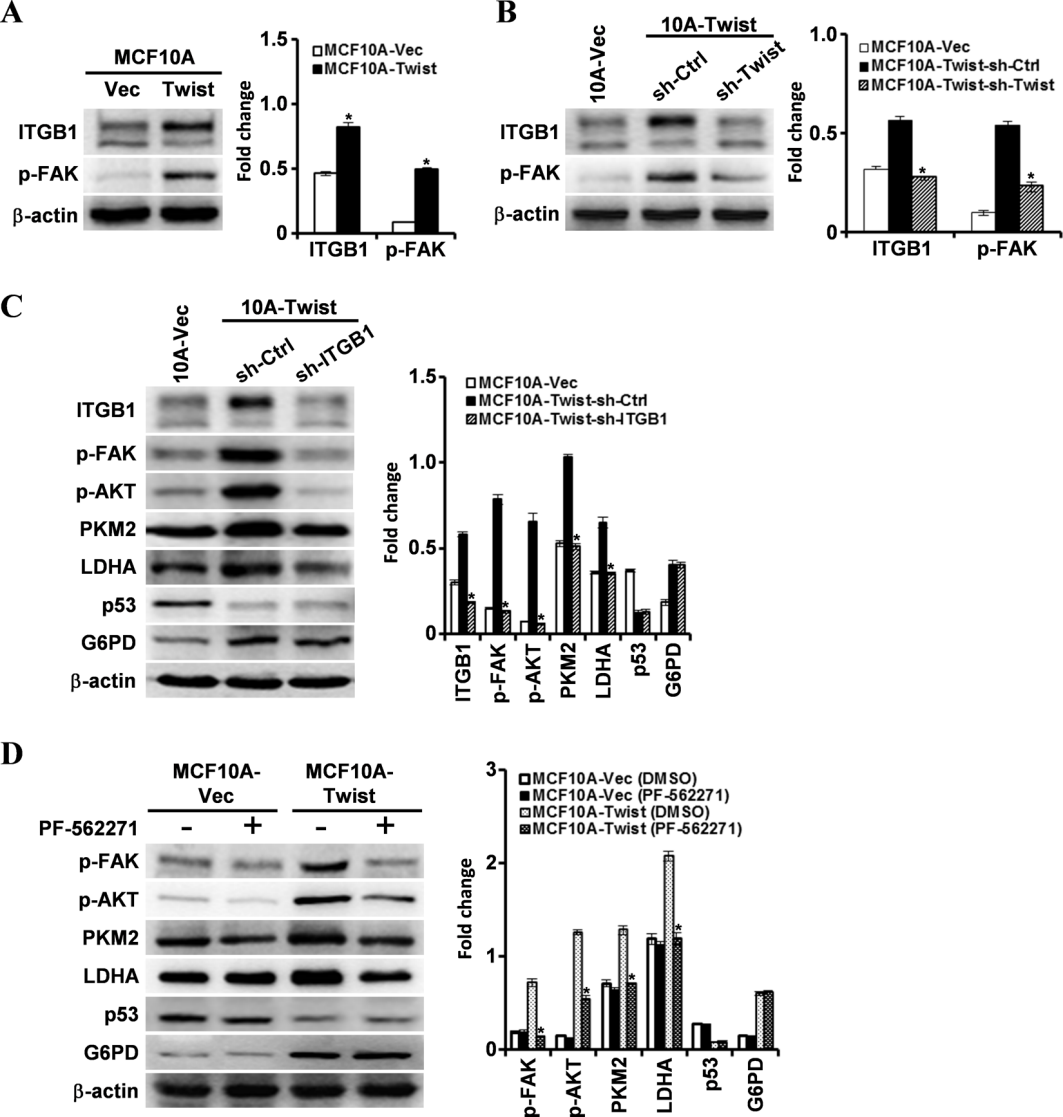
Twist activates the FAK pathway and its downstream PI3K/AKT through upregulating β1-integrin The protein levels were determined by Western blotting analysis in the indicated cells, and β-actin was used as an internal control. (*P < 0.05). **A.** The β1-integrin (ITGB1) and p-FAK expression in MCF10A-Vector cells and MCF10A-Twist cells. **B.** The levels of β1-integrin (ITGB1) and p-FAK in MCF10A-Vector cells, MCF10A-Twist-sh-Ctrl cells and MCF10A-Twist-sh-Twist cells. **C.** The β1-integrin (ITGB1), p-AKT, PKM2, LDHA, p53, and G6PD expression in MCF10A-Vector cells, MCF10A-Twist transfected with control shRNA (MCF10A-Twist-sh-Ctrl), MCF10A-Twist transfected with β1-integrin shRNA (MCF10A-Twist-sh-ITGB1). **D.** After treatment with PF-562271 (FAK inhibitor), expression of p-FAK, p-AKT, PKM2, LDHA, p53, and G6PD in MCF10A-Vector and MCF10A-Twist cells was determined.

β1-integrin is known to activate FAK in both normal and transformed cells [31, 32]. β1-integrin level was found to be more than 3 times higher in MCF10A-Twist cells compared with MCF10A-Vector cells according to our proteomic analysis (Data not shown). Indeed, using western blotting, high levels of β1-integrin and phosphorylated FAK (p-FAK) were detected in MCF10A-Twist cells (Fig. 5A) and Twist-positive BT549 cells (Supplemental Fig. 6A). Knockdown of Twist in MCF10A-Twist and BT549 cells decreased the levels of β1-integrin and p-FAK in MCF10A-Twist-sh-Twist and BT549-sh-Twist cells (Fig. 5B and Supplemental Fig. 6B). To further investigate whether β1-integrin/FAK signaling pathway is implicated in promoting EMR, the expression of the EMR-associated PKM2 and LDHA, and the EMR biomarker of glucose consumption and lactate production were determined. Silence of β1-integrin in MCF10A-Twist and BT549 cells resulted in down-regulation of p-FAK, p-AKT, PKM2 and LDHA. p53 and G6PD expression did not show significant changes (Fig. 5C and Supplemental Fig. 6C). After PF-562271 (an inhibitor of FAK) treatment, the expression of p-FAK, p-AKT, PKM2 and LDHA, but not p53 and G6PD was decreased in MCF10A-Twist (Fig. 5D) and BT549 cells (Supplemental Fig. 6D). Correspondingly, glucose consumption and lactate production in MCF10A-Twist (Supplemental Fig. 7A–7B) and BT549 (Supplemental Fig. 7D–7E) were also decreased. Mitochondrial mass in MCF10A-Twist and BT549 treated with PF-562271 was higher than that in their control cells (Supplemental Fig. 7C and 7F). These data suggest that Twist activates β1-integrin/FAK signaling pathway and its downstream PI3K/AKT signaling in MCF10A-Twist and BT549 cells, and thus promotes EMR of these cells.

### Twist directly represses the transcription of p53

**Figure 6:**
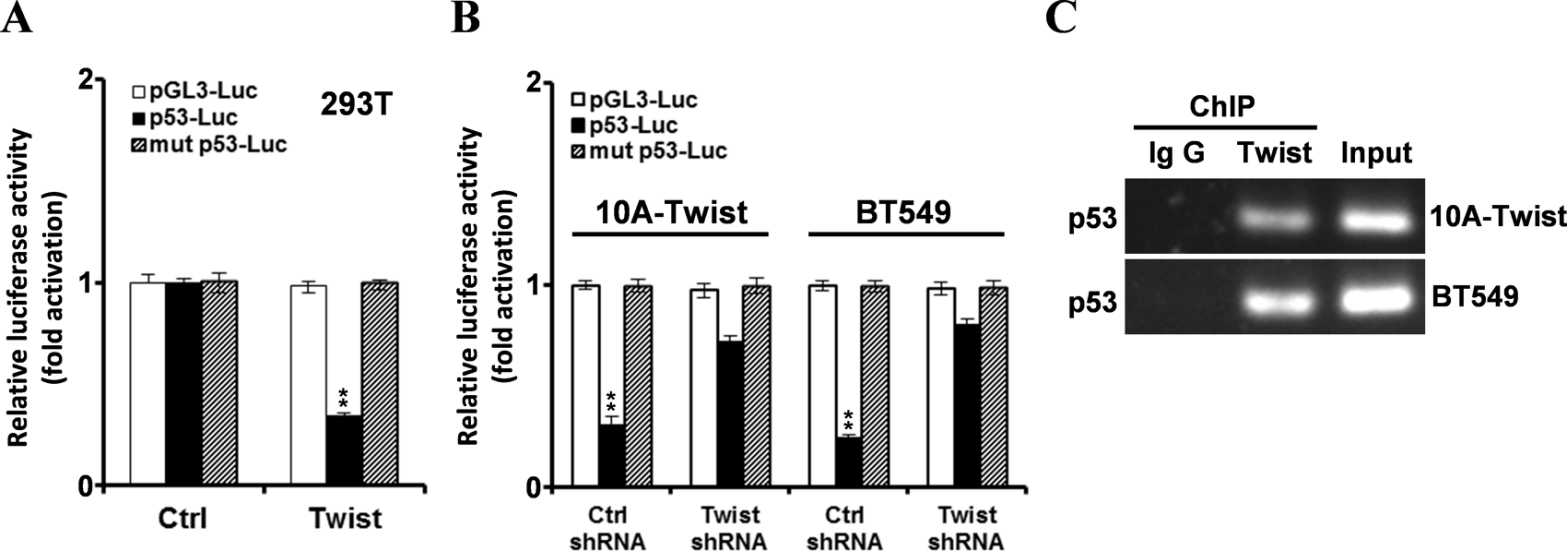
Twist inhibits p53 expression via binding to the E-box of p53 promoter **A.** Luciferase assays were conducted in HEK293T cells co-transfected pGL3-Luc or pGL3-p53-Luc or pGL3-mut p53-Luc report plasmid with Twist or its control vector respectively. After 30 h, the luciferase activity was determined. The experiments were repeated 3 times. (**P < 0.01). **B.** MCF10A-Twist cells and BT549 cells was co-transfected with pGL3-Luc or pGL3-p53-luc or pGL3-mut p53-luc report constructer in the presence of Twist shRNA or control shRNA respectively, and about 60 h, the luciferase activity was determined. The experiments were repeated 3 times. (**P < 0.01). **C.** MCF10A-Twist cells and BT549 cells were processed for ChIP analysis using c-Myc (MCF10A-Twist cells) or Twist (BT549 cells) antibody for immunoprecipitation followed by semi-quantitative PCR with p53 promoter-specific primers. IgG was used as a control antibody.

Previous study has demonstrated that Twist can bind to the E-box of p53 to repress gene transcription of p53 [33]. To verify this repression of p53 in Twist-positive cells, luciferase assays and chromatin immunoprecipitation assays were conducted. As shown in Fig. 6A, after transient transfection of p53-Luc, mut-p53-Luc and Twist in HEK293T cells, ectopic expression of Twist dramatically inhibited p53 promoter activity. In contrast, Twist did not affect the activity of E-box mutant p53 promoter. This was further confirmed by transfection of p53-Luc or mutant p53-Luc into MCF10A-Twist, Twist-positive BT549 and their Twist-silenced cells. Knockdown of Twist in MCF10A-Twist and BT549 cells significantly increased the activity of p53 promoter but not the E-box mutant p53 promoter (Fig. 6B). Binding of Twist to the p53 promoter was clearly detected in MCF10A-Twist cells and BT549 cells (Fig. 6C). Overall, these results demonstrate that Twist inhibits p53 expression by binding to the E-box of p53.

### EMR in MCF10A-Twist and Twist-positive breast cancer cells enhances cell migration

**Figure 7:**
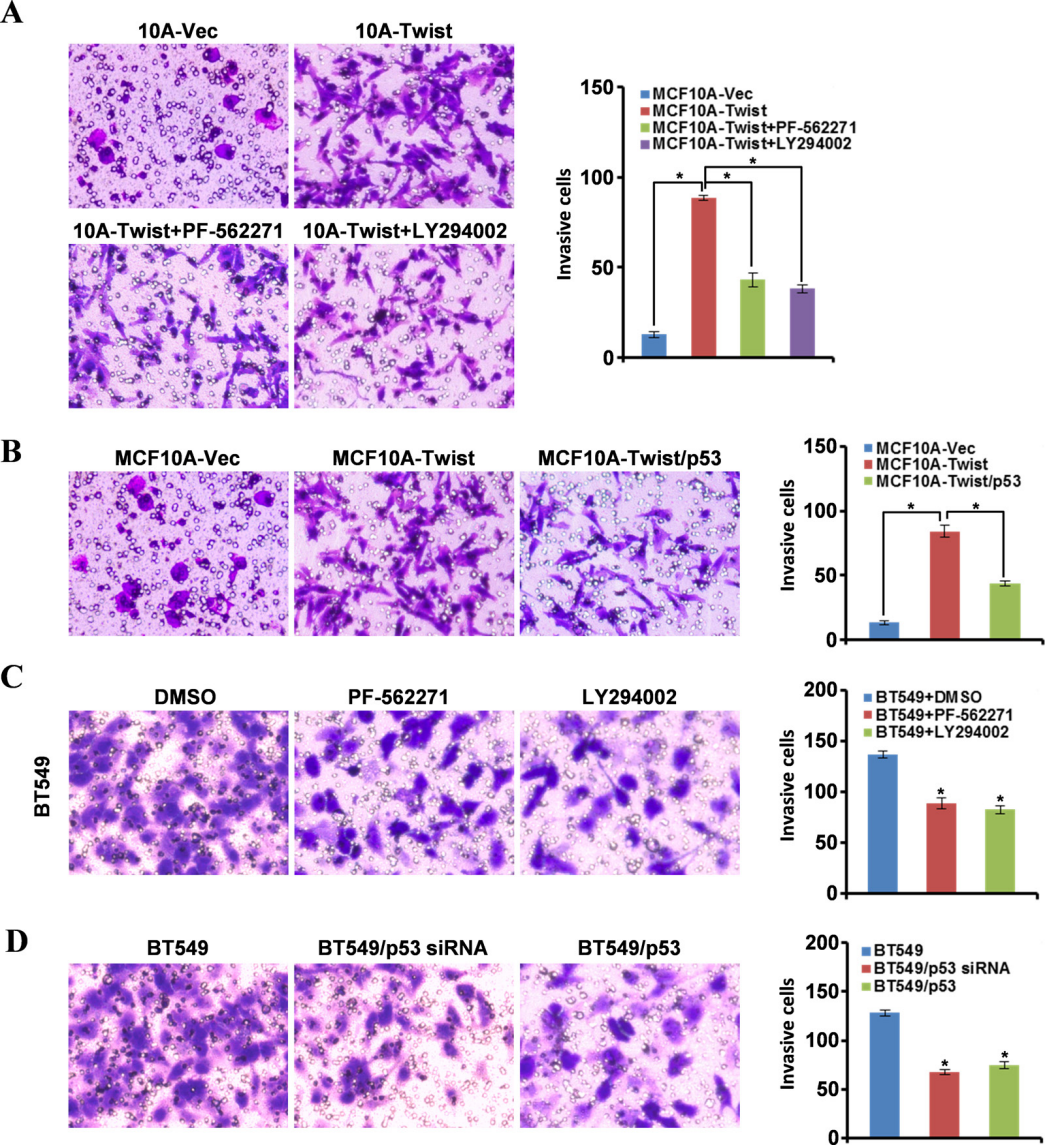
Energy metabolism reprogramming enhances the ability of cell migration **A, C.** Transwell assays were employed to test the cell migratory capacity of MCF10A-Twist, BT549 cells under blockage of FAK activity by PF-562271, or blockage of the PI3K/AKT/mTOR pathway by LY294002 (*P < 0.05). **B, D.** The migration potential of MCF10A-Twist and BT549 cells was examined by Transwell Chamber. MCF10A-Twist transfected with pCMV-HA-p53 (MCF10A-Twist/p53), BT549 transfected with p53 siRNA (BT549/p53 siRNA) or with pCMV-HA-p53 (BT549/p53) (*P < 0.05).

Finally, we asked whether EMR could lead to enhanced cell migration ability. Compared with MCF10A-Vector, MCF10A-Twist showed stronger migratory capacity (Fig. 7A, left upper). After treatment with PF-562271 or LY294002 (Fig. 7A, left down) or restoration of p53 expression in MCF10A-Twist (Fig. 7B), cell migration ability was reduced. Similar findings were confirmed in BT549 cells (Fig. 7C–7D). Specially, silence of endogenous mutant p53 in BT549 using its siRNA or overexpression of wild-type p53 in BT549, cell migration was notably decreased. Thus, blockage of β1-integrin/FAK-PI3K/AKT signaling or rescue of functional p53 expression in MCF10A-Twist and Twist-positive BT549 cells inhibits EMR and impedes cell migration. Taken together, these data suggest a close association between EMR induced by Twist and cancer cells' ability of migration and metastasis.

## DISCUSSION

In this study, we evaluated whether EMR is regulated by Twist in MCF10A-Twist and Twist-positive breast cancer cells. We found that the expression of Twist resulted in an altered energy metabolic phenotype of MCF10A-Twist and Twist-positive breast cancer BT549 cells. Compared with MCF10A-Vector, MCF10A-Twist consumed more glucose, produced more lactate and had lower mitochondrial mass. Furthermore, hypoxia treatment potentiated these changes. Consistent with these findings, our data suggest that loss of Twist expression reversed the altered energy metabolic phenotype of MCF10A-Twist and BT549 cells. Our data strongly indicate that Twist is capable of triggering EMR in breast cancer cells.

Using cDNA microarray and proteomics analysis, we found that there was altered expression of a set of glucose metabolism-related genes in MCF10A-Twist cells compared to that in its control cells. The mRNA expression of G6PD, PKM2, LDHA, PGK1, ENO1 and TPI1 was increased in MCF10A-Twist. Protein expression of PKM2, LDHA and G6PD, key genes related to EMR, was further confirmed in MCF10A-Twist. Moreover, silencing Twist by RNA interference diminished the expression of PKM2, LDHA and G6PD in MCF10A-Twist and BT549. PKM2, the M2 isoform of pyruvate kinase (PK), is expressed in highly proliferating cells, including in all cancer cell lines and tumors studied to date [34, 35]. Previous researches have shown that PKM2 expression is essential for aerobic glycolysis of cancer cells and tumor growth [36–38]. Both *in vivo* and *in vitro* experiments have implicated that high expression of PKM2 promotes Warburg effect and provides a selective growth advantage for tumor cells [36]. LDHA, which executes the final step of aerobic lactate production, is elevated in many human cancers and has been linked to tumor growth, maintenance, and invasion [39–42]. Expression of LDHA can be affected by PKM2, GLUT1 and LDHA. PKM2 is essential for the EGFR induced Warburg effect [43]. G6PD is the first and rate-limiting enzyme of PPP [44] and plays a critical role in tumorigenesis [45, 46]. The activity of G6PD is negatively regulated by p53, which can block the formation of active dimers of G6PD [25]. Our data suggest that the overexpression of Twist in breast cancer cells may result in EMR through up-regulating of PKM2, LDHA, and G6PD. In addition, we also found that p-AKT was increased and p53 was repressed by Twist in MCF10A-Twist. Our previous studies have demonstrated that a set of multiple canonical signal pathways including PI3K/AKT and p53 signaling pathways are altered in MCF10A-Twist (unpublished data). PI3K/AKT pathway is closely related to glucose metabolism and cell invasion in tumor. Activation of PI3K/AKT signaling resulted in an enhanced anaerobic glycolysis [47]. AKT may constitute a “Warburg kinase”, as AKT hyper-activation increases tumor cells glucose uptake and glycolysis [20].

In the past few years, it became clear that the tumor suppressor p53 can also directly control metabolic traits of cells. p53 plays a role in promoting oxidative phosphorylation and inhibiting glycolysis [21, 48, 49]. Moreover, the p53 protein binds to G6PD and prevents the formation of the active dimer, thus inhibiting the PPP [25]. Consistently, p53 inactivation in tumor cells likely accelerates glucose consumption via activation of the PPP and glycolysis. Therefore we suppose that PI3K/AKT and p53 signaling pathways may be responsible for EMR induced by Twist in MCF10A-Twist cells and Twist-positive breast cancer cells.

Our data has demonstrated that Twist can activate FAK and its downstream PI3K/AKT pathway through upregulating the expression of β1-integrin. In order to demonstrate our hypothesis, β1-integrin/FAK/PI3K/AKT pathway was blocked using a chemical inhibitor or RNAi interference. Our results indicated that blockage of β1-integrin/FAK-PI3K/AKT pathway partly reversed the phenotype of EMR induced by Twist and reduced the expression of PKM2 and LDHA in MCF10A-Twist and BT549. Overall, these findings suggest that β1-integrin/FAK/PI3K/AKT pathway is involved in EMR induced by Twist in MCF10A-Twist and Twist-positive breast cancer cells. To further uncover the molecular mechanism of how PKM2 was regulated by PI3K/AKT pathway, mTOR specific siRNA was used to inhibit mTOR expression. mTOR, a downstream effector of AKT, has been identified as a central activator of Warburg effect by inducing PKM2 and other glycolytic enzymes under normoxic conditions [22]. We found that PKM2 and LDHA were down regulated and glucose consumption and lactate production were decreased after transfected with mTOR siRNA. These results suggest that β1-integrin/FAK/PI3K/AKT pathway upregulates PKM2 through mTOR in MCF10A-Twist and Twist-positive breast cancer cells. Besides, glucose consumption and lactate production were decreased, and mitochondrial mass was increased in MCF10A-Twist and BT549 cells treated with FAK inhibitor. Taken together, Twist promotes glycolysis via activation of β1-integrin-FAK-PI3K-AKT-mTOR axis in MCF10A-Twist and BT549 cells. Furthermore, previous studies have proved that Twist directly interacts with the DNA binding domain of p53 to suppress its DNA-binding and transcriptional activity and Twist may affect p53 protein level indirectly through modulation of the ARF/MDM2/p53 pathway [50]. Moreover, Twist also enhances MDM2-mediated degradation of p53 through an E-box-independent mechanism. Twist binds p53 C terminus through the Twist box and the interaction is critical for Twist inhibition of p53 [51]. Interestingly, our results indicate that overexpression of Twist in MCF10A cells results in down-regulation of wild-type p53, which relieves the inhibition effect of wild-type p53 on G6PD and leads to up-regulation of G6PD. We subsequently demonstrated that Twist can directly inhibit the expression of p53 by binding to the E-box of human p53 gene promoter, which was consistent with previous studies [33]. We further demonstrated that overexpression of wild-type p53 in MCF10A-Twist decreased the expression of G6PD. Nevertheless, BT549 expresses high level of mutant p53 (R249S) [28] and it has little influence on G6PD expression. Overexpression of wild-type p53 decreased the G6PD expression in BT549. Our data further proved that p53 inactivation caused by mutation made it lose the ability to regulate G6PD. In addition, we found that overexpression of wild-type p53 inhibited the mTOR expression and decreased the glucose consumption and lactate production in MCF10A-Twist and BT549. These results suggest that Twist also relieves the inhibition of wild-type p53 on mTOR pahway and thus promotes glycolysis. The alterations in energy metabolism could sustain the growth of breast cancer cells and promote the migration of breast cancer cell by providing energy for cells. In summary, our findings suggest that Twist may activate PPP and glycolysis pathway by down-regulating wild-type p53 or inactivating of wild-type p53, thereby promoting EMR and contributing to Warburg effect in MCF10A-Twist and Twist-positive breast cancer cells.

To our knowledge, this is the first time to study the effect of Twist on EMR in breast cancer cells. Our results indicate that Twist can induce EMR and cell migration by activating the β1-integrin/FAK/PI3K/AKT/mTOR pathway and repressing the p53 pathway in MCF10A-Twist cells and Twist-positive breast cancer cells.

In conclusion, this study sheds light on the mechanisms of EMR regulation in breast cancer cells and provides new avenues for breast cancer treatment.

## MATERIALS AND METHODS

### Reagents, plasmids, and cell culture

Most reagents used in this work are commercial products. Cholera toxin was obtained from Sigma (St. Louis., MO, USA). Epidermal Growth Factor was from Life Technologies (Carlsbad, CA., USA). Mito-Tracker Green was from Beyotime (Haimen, Jiangsu, China). Glucose Assay Kit was from Rsbio (Shanghai, China). Lactate Assay Kit was from Njjcbio (Nanjing, Jiangsu, China). LY294002 and PF-562271 were obtained from Selleck Chemicals (Houston, TX, USA). Lipofectamine™ 2000 was purchased from Life Technologies (Carlsbad, CA., USA). Antibodies against LDHA, G6PD, mTOR and p-FAK (Y397) were obtained from Bioworld (Nanjing, Jiangsu, China). Antibody against β1-integrin was from Abcam (Cambridge, UK). Antibodies against PKM2, p-AKT (S473), and p53 were from Cell Signaling Technology (Beverly, MA, USA). β-actin antibody, goat antimouse IgG-HRP, and goat antirabbit IgG-HRP were obtained from Santa Cruz Biotechnology (Santa Cruz, CA, USA). The retroviral expression vectors encoding c-Myc-tagged Twist was described previously [19]. pCMV-HA-p53 was constructed by inserting human p53 cDNA into pCMV-HA (Clontech, PaloAlto, CA, USA). The p53 promoter (−291 to +71 region) was inserted into the pGL3-basic vector (Promega, Madison, WI, USA) at KpnI/XbaI sites to construct pGL3-p53 luciferase reporter plasmid. The E-box site of the p53 promoter was mutated (from 5′-CAGCTG-3′ to 5′-TGGCTG-3′) using the QuikChange Site-directed mutagenesis kit (Stratagene, La Jolla, CA), and then the E-box site-mutated p53 promoter was cloned into the pGL3-basic vector (named as pGL3-mut p53 luciferase reporter here after). All constructs were verified by sequencing.

The immortalized normal human mammary epithelial cell line MCF10A, human breast cancer cell lines MCF7 and BT549, and HEK293T were obtained from ATCC (Rockville, MD, USA). MCF10A-Vector and MCF10A-Twist cells were established as previously described [19]. MCF10A, MCF10A-Vector, and MCF10A-Twist were cultured in DMEM/F-12 medium. MCF7 and BT549 cells were cultured in RPMI-1640 medium supplemented with 10% FBS. Hypoxia treatment of cells was performed in a tri-gas incubator (Thermo, USA) flushed with a gas mixture of 1% O_2_, 5% CO_2_ and 94% nitrogen. For chemical treatment, cells were cultured in 12-well plates for 24 h. Then the cultured medium was replaced by fresh medium containing 50 μM LY294002 or vehicle for 6 h, or by fresh medium containing 10 μM PF-562271 or vehicle for 24 h.

### Luciferase assays

For the luciferase reporter assay, HEK293T cells were co-transfected with pGL3-Luc, pGL3-p53-Luc, or pGL3-mut p53-Luc and Twist construct or its control vector. MCF10A-Twist and BT549 cells were transfected with pGL3-Luc, pGL3-p53-Luc, or pGL3-mut p53-Luc and Twist shRNA or its control shRNA using Lipofectamine™ 2000. The Renilla luciferase was as internal control. After transfection, cells were incubated for 30 h (293T cells) or 60 h (MCF10A-Twist and BT549 cells) and luciferase activity was examined by using the luciferase assay system (Promega, Madison, WI, USA). The relative firefly luciferase activity was calculated by normalizing transfection efficiency according to the Renilla luciferase activity.

### RNA extraction and qRT-PCR

Total RNA was isolated using TRIzol reagent according to the manufacturer's specifications. Quantitative real-time PCR (qRT-PCR) was performed as described previously [52]. The primers are listed in Supplemental Table 1.

### RNA interference

The lentivirus vector containing a cytomegalovirus (CMV)-driven GFP reporter and a U6 promoter upstream of the cloning sites (Age I and EcoR I) was used for cloning small hairpin RNAs (shRNAs). The lentiviral expression vector of Twist1 shRNA and its infective lentivirus were obtained from GenePharma (Shanghai, China). The target sequence for Twist1 is 5′-AAGCTGAGCAAGATTCAGACC-3′. The lentiviral expression vector of β1-integrin shRNA and its infective lentivirus were obtained from GeneChem (Shanghai, China). The target sequences for β1-integrin are 5′-CCGGGAGGAAATGGTGTTTGCAAGTTTCAAGAG AACTTGCAAACACCATTTCCTCTTTTTG-3′ (forward), 3′-CTCCTTTACCACAAACGTTCAAAGTTCTCTTGAA CGTTTGT GGTAAAGGAGAAAAACTTAA-5′ (reverse). The control shRNA sequence is 5′-TTCTCCGAA CGTGTCACGT-3′. Cell infection with lentivirus was carried out for 12 h, and then the culture medium was replaced by fresh growth medium. The efficiency of gene knockdown was determined by qRT-PCR and western blotting analysis.

The mTOR or p53 siRNA (Ribobio, Guangzhou, China) was transiently transfected into cells using Lipofectamine™ 2000 (Life Technologies, Carlsbad, CA., USA) following the manufacturer's protocol. The target sequences for mTOR siRNA are 5′-GGAGUCUACUCGCUUCUAUTT-3′ (A) and 5′-AUAGAAGCGAGUAGACUCCTC-3′ (B). The target sequences for p53 siRNA are 5′-AAGAAA TTTGCGTTTGGAGTA-3′ (A), and 5′-AAGGAAGACTCCAGTGGTAAT-3′ (B). The control siRNA sequence is 5′-AAGG TGTCAGAAACTGACGAT-3′.

### Detection of mitochondrial mass, glucose consumption, and lactate production

Mitochondrial mass was measured by staining with Mito-Tracker Green. Cells attached to the cover glass were washed and incubated with pre-warmed Mito-Tracker staining solution with a final concentration of 100 nM at 37°C in the dark. Staining solution was removed after 30 min and replaced by pre-warmed fresh culture medium. Afterwards, the cells were observed under a fluorescence microscope (Nikon Eclipse 80i, Tokyo, Japan).

For detection of glucose, lactate concentration, cells were seeded in 12-well plates at appropriate concentrations. When cell confluence was 60%–70%, the culture medium was replaced by fresh growth medium. After different treatment, the supernatant of culture medium was collected for measurement of glucose and lactate concentrations. The levels of glucose were determined using the Glucose Assay Kit and the levels of lactate were determined using the Lactate Assay Kit under microplate reader (BioTek, Winooski, Vermont, USA) according to their respective manufacturer's protocols. At the same time, the number of cells in each well was counted. Glucose consumption and lactate production were normalized to cell number, respectively.

### Mitochondrial detection by TEM

MCF10A-Twist and MCF10A-Vector cells were cultured under normoxic or hypoxic conditions for the indicated time. Cells were then harvested and fixed in glutaraldehyde for 2 h, followed by 1% osmium tetroxide for 2 h at 4°C. After being dehydrated in ethanol and acetone, the cells were embedded in epoxy resin and polymerized at 60°C. Ultrathin sections with the thickness of 60 nm were prepared and stained with uranyl acetate and lead citrate. Slides were observed under TEM (Hitachi-7500, Japan) and photographed using a CCD camera (Gatan-780CCD, USA).

### Western blotting analysis

Western blotting was performed as described previously [53]. The specific primary antibodies used in this study were PKM2 (1:1000), LDHA (1:2000), G6PD (1:1000), p-AKT (1:1000), mTOR (1:1000), p-FAK (1:500), β1-integrin (1:1000), Twist (1:100) and p53 (1:500). The appropriate HRP-labeled secondary antibodies were subsequently applied and signals were then visualized by enhanced chemiluminescence reagents (ECL, Millipore, MA, USA).

### Chromatin immunoprecipitation assays

Chromatin immunoprecipitation (ChIP) assays were performed as previously described [54] and anti-c-Myc antibody (Cell Signaling Technology, Beverly, MA, USA) or anti-Twist antibody (Abcam Cambridge, UK) was used to precipitate the protein/DNA complex. DNA fragments were extracted and amplified by PCR. The human p53 promoter-specific primers used in PCR were 5′- CATTTTAACTGATGAGAAGAAAGGA-3′ (forward), 5′-GGACAGTCGCCATGACAAG-3′ (reverse). Input DNA was analyzed in parallel in order to normalize the results.

### Transwell migration assays

The migration abilities of cells were measured by transwell assays with modified Boyden chambers containing polycarbonate filters (Millipore, MA, USA) according to the manufacturer's instructions. Cells were seeded into the upper Boyden chambers in serum-free medium and allowed to invade towards 10% FBS medium for 12 to 48 h. Cells that remained on top of the filter were removed using a cotton applicator and the invaded cells at the opposite side of the filter were fixed with 4% paraformaldehyde, and stained with 0.5% crystal violet before counting under a microscope.

### Statistical analysis

Data represent at least three separate experiments done in triplicate (mean ± standard error). The independent Student's *t*-test was calculated to compare the results between two groups. The analysis of variance was applied to compare the multiple groups. All statistical analyses were performed using the SPSS software system. *P*-values < 0.05 were considered statistically significant.

## SUPPLEMENTARY FIGURES AND TABLES


