# Supplementary material for: Twist promotes reprogramming of glucose metabolism in breast cancer cells through PI3K/AKT and p53 signaling pathways
Source: Oncotarget. 2015 Jul 27;6(28):25755–69. doi: 10.18632/oncotarget.4697 (PMC4694864; doi:10.18632/oncotarget.4697)
Supplement: Supplementary file 1 [file oncotarget-06-25755-s001.pdf]

SUPPLEMENTARY FIGURES AND TABLE

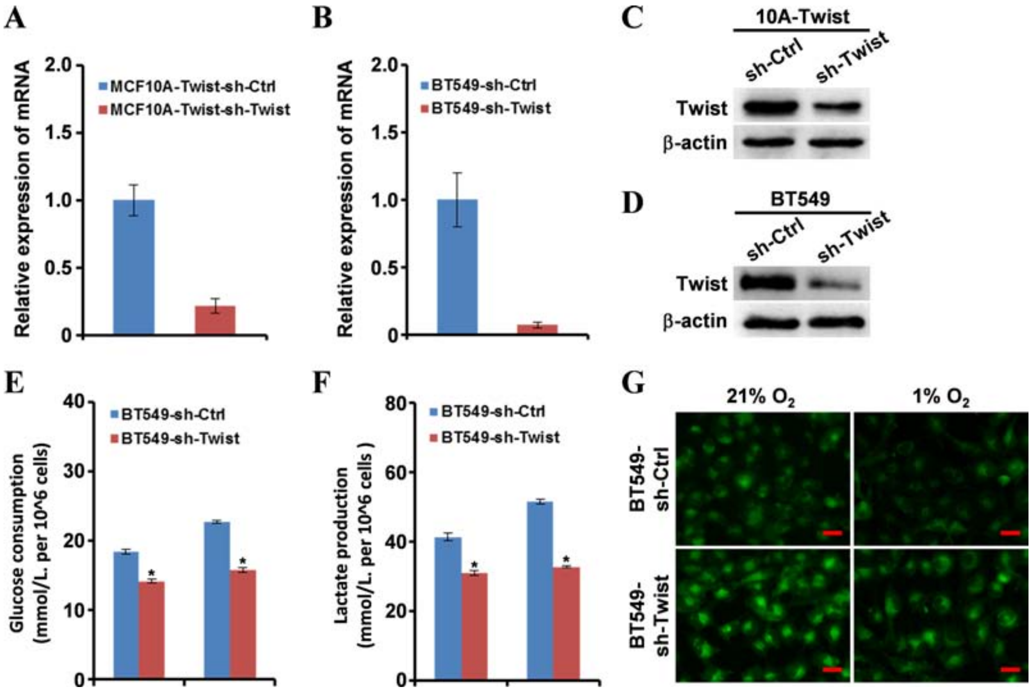

Supplementary Figure S1: Loss of Twist expression reverses the energy metabolic phenotype in Twist-positive breast cancer cells. A, B. qRT-PCR was used to analyze the Twist expression in MCF10A-Twist (A) or BT549 (B) with silenced Twist (MCF10A-Twist-sh-Twist, BT549-sh-Twist) and control cells (MCF10A-Twist-sh-Ctrl, BT549-sh-Ctrl). **C, D.** Western blotting analysis was used to determine the Twist expression in MCF10A-Twist-sh-Twist and MCF10A-Twist-sh-Ctrl (C), or BT549-sh-Twist and BT549-sh-Ctrl cells (D). β-actin was used as an internal control. **E.** Fluorescence microscope analysis of mitochondrial mass in BT549-sh-Twist and BT549-sh-Ctrl cells after Mito-Tracker Green staining. Magnification, x200. Scale bars, 100 μm.

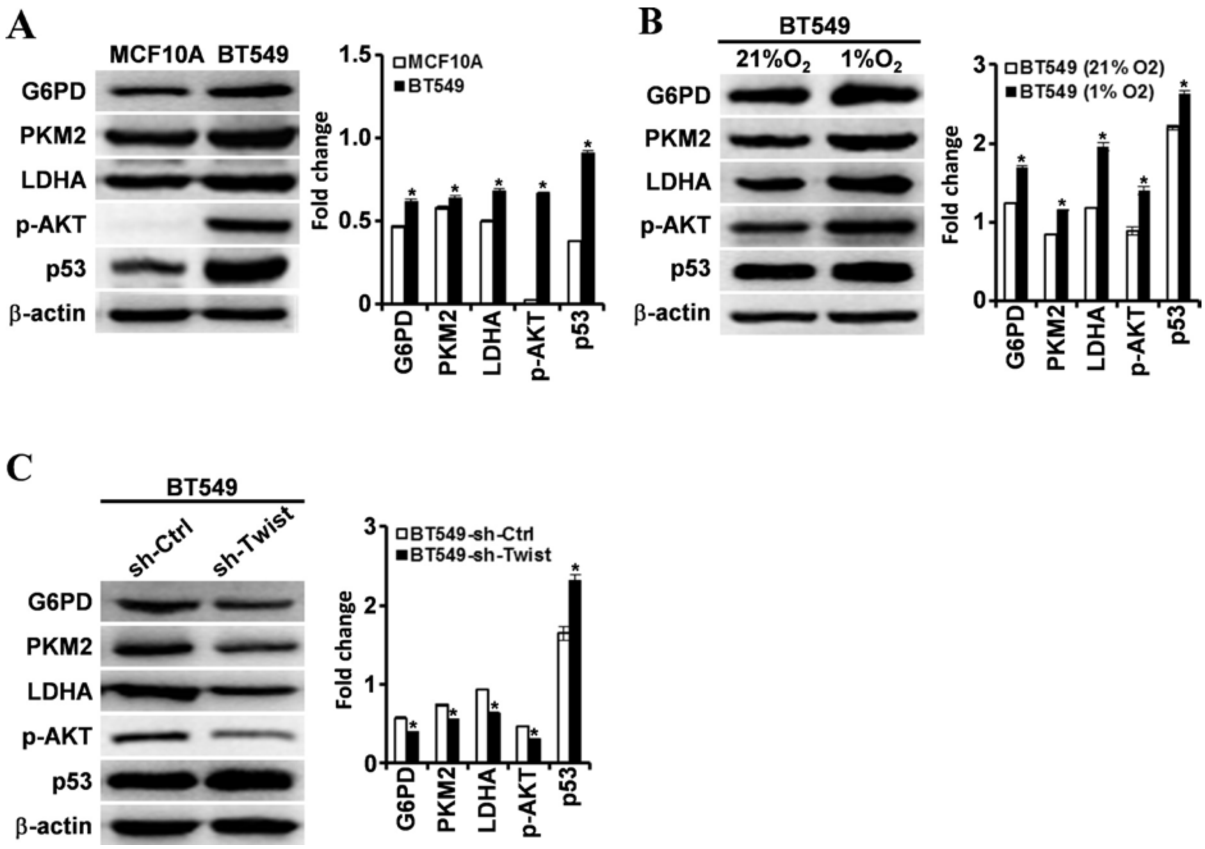

Supplementary Figure S2: Twist changes the expression of genes linked to energy metabolism in Twist-positive breast cancer cells. A. The expression of G6PD, PKM2, LDHA, p-AKT, and p53 in MCF10A and BT549 cells was determined by western blotting. β-actin was used as an internal control. (*$P < 0.05$ versus control). **B.** Effect of hypoxia treatment on the expression of key genes related to cell energy metabolism in BT549 cells. β-actin was used as an internal control. (*$P < 0.05$ versus control). **C.** The expression of key genes associated with cell energy metabolism in BT549-sh-Twist and BT549-sh-Ctrl cells was analyzed by western blotting. β-actin was used as an internal control. (*$P < 0.05$ versus control).

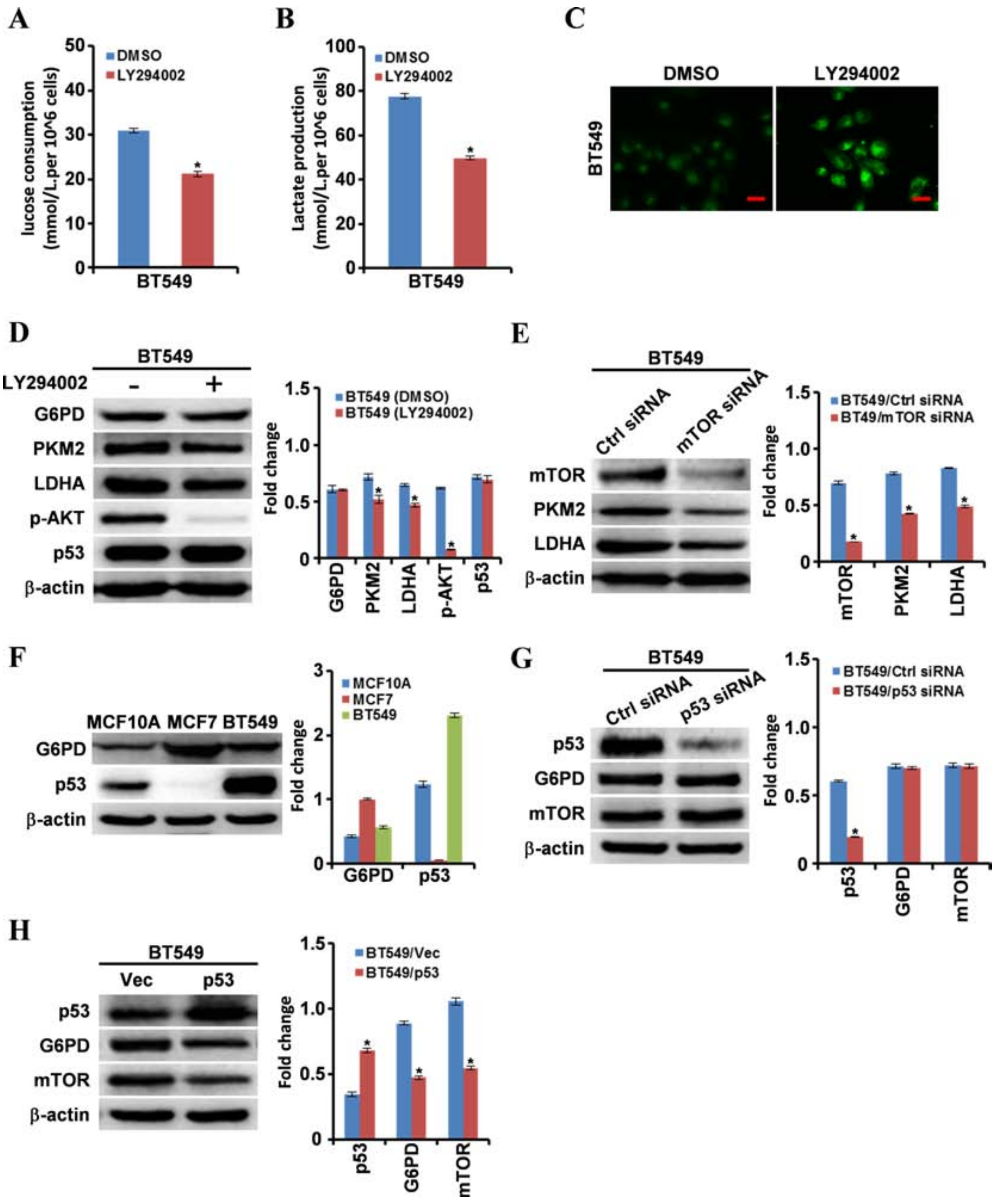

Supplementary Figure S3: Molecular mechanisms of energy metabolism reprogramming in Twist-positive breast cancer cells. A, B. After treated with or without LY294002, glucose consumption and lactate production of BT549 cells were detected (*$P < 0.05$ versus control). **C.** Fluorescence microscope analysis of mitochondrial mass in BT549 cells treated with or without LY294002 by Mito-Tracker Green staining (Magnification, x200. Scale bars, 100 µm). **D, E.** The protein levels were determined using western blotting. β-actin was used as an internal control. (*$P < 0.05$ versus control). Levels of G6PD, PKM2, LDHA, p-AKT, and p53 in BT549 cells treated with or without LY294002 (D); and (E) mTOR, PKM2 and LDHA expression in BT549 transfected with control siRNA or mTOR siRNA. **F.** Levels of p53 and G6PD in MCF10A, MCF7 and BT549 were detected by western blotting. β-actin was used as an internal control. **G, H.** Western blotting was applied to detect the p53, mTOR and G6PD levels. β-actin was used as an internal control. (*$P < 0.05$). (G) BT549 transfected with control siRNA or p53 siRNA; and (H) BT549 transfected with pCMV-HA-p53 (BT549/p53) or its control vector (BT549/Vec).

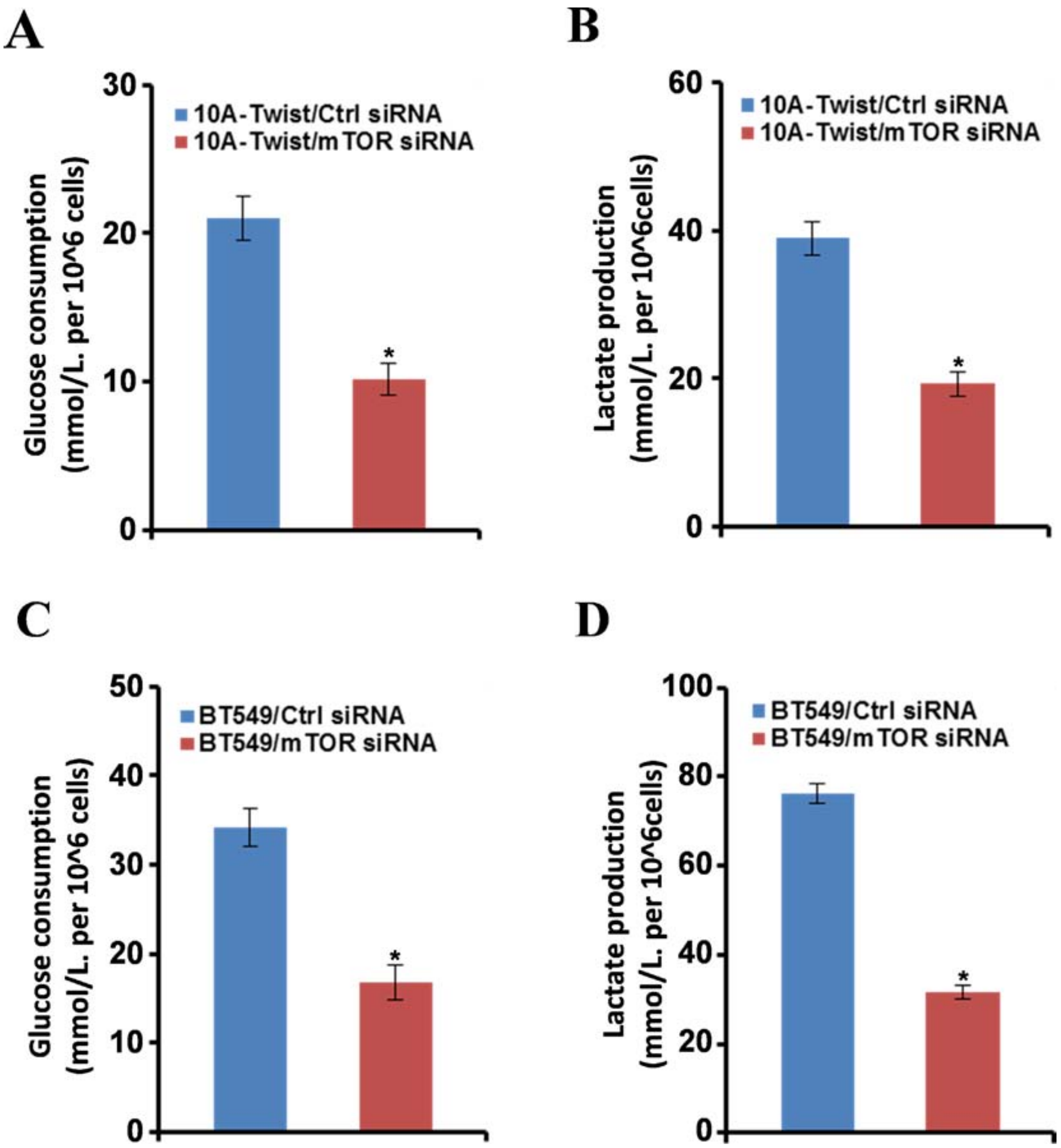

Supplementary Figure S4: The changes in the phenotype of energy metabolism reprogramming in MCF10A-Twist cells or BT549 cells transfected with mTOR siRNA. A, B. After transfected with mTOR siRNA or its control siRNA, glucose consumption and lactate production of MCF10A-Twist cells were detected. *$P < 0.05$ versus control. **C, D.** After transfected with mTOR siRNA or its control siRNA, glucose consumption and lactate production of BT549 cells were detected. *$P < 0.05$ versus control. MCF10A-Twist transfected with control siRNA (MCF10A-Twist/Ctrl siRNA) or mTOR siRNA (MCF10A-Twist/mTOR siRNA), BT549 transfected with control siRNA (BT549/Ctrl siRNA) or mTOR siRNA (BT549/mTOR siRNA).

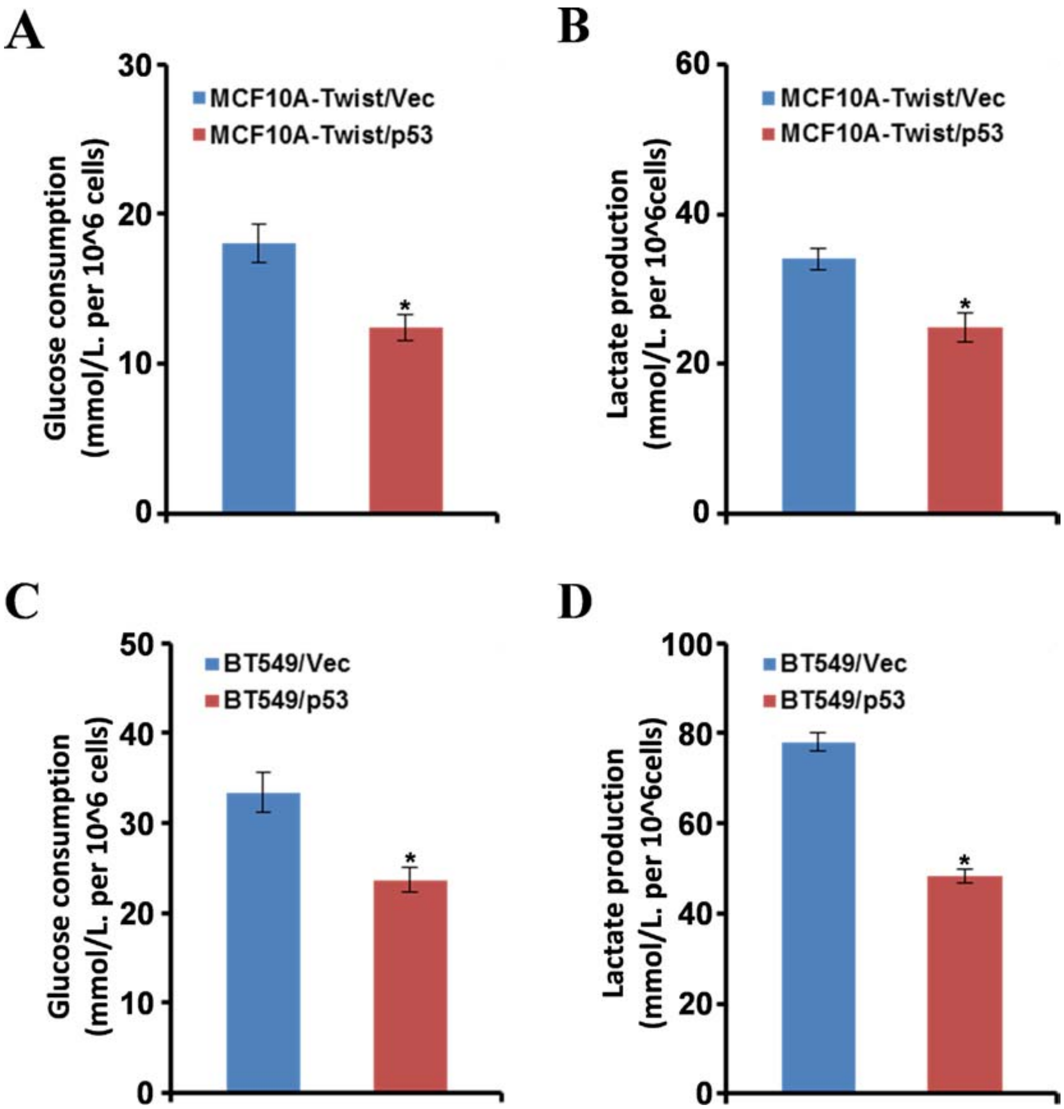

Supplementary Figure S5: The changes in the phenotype of energy metabolism reprogramming in MCF10A-Twist cells or BT549 cells transfected with pCMV-HA-p53. A, B. After transfected with pCMV-HA-p53 or its control vector, glucose consumption and lactate production of MCF10A-Twist cells were detected. $^*P < 0.05$ versus control. **C, D.** After transfected with pCMV-HA-p53 or its control vector, glucose consumption and lactate production of BT549 cells were detected. $^*P < 0.05$ versus control. MCF10A-Twist transfected with pCMV-HA-p53 (MCF10A-Twist/p53) or its control vector (MCF10A-Twist/Vec), BT549 transfected with pCMV-HA-p53 (BT549/p53) or its control vector (BT549/Vec) ($^*P < 0.05$).

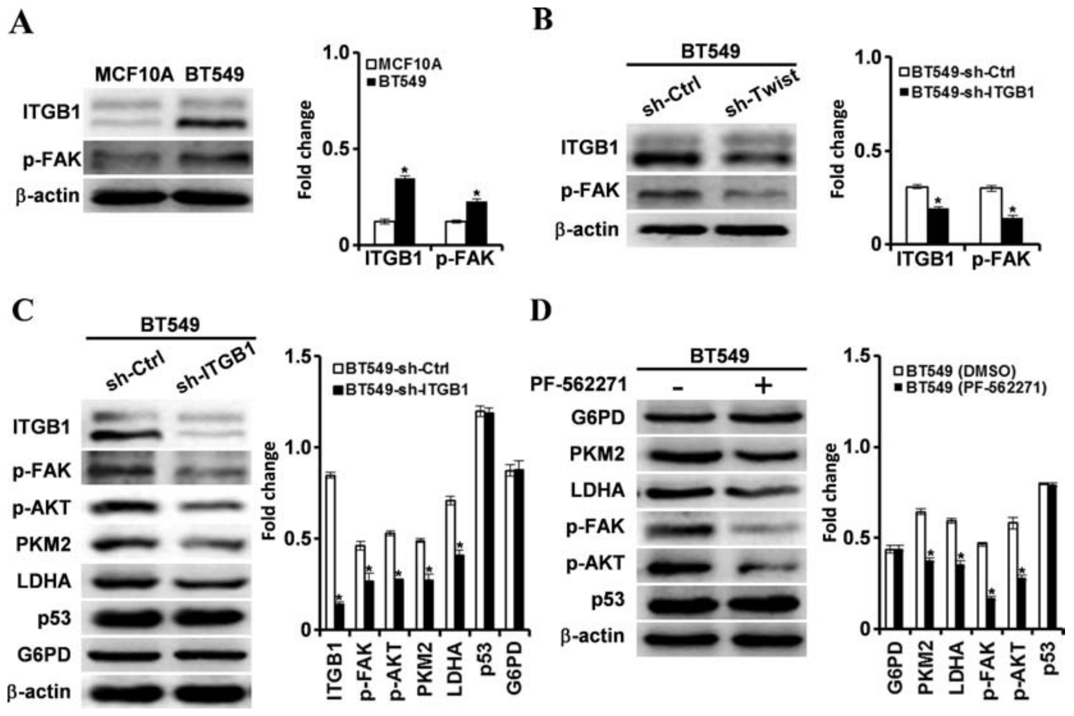

Supplementary Figure S6: Twist activates FAK pathway and its downstream PI3K/AKT pathway through upregulating β1-integrin and changes the expression of genes related to cell energy metabolism in BT549 cells. The expression of genes was analyzed by western blotting, and β-actin was used as an internal control. $^*P < 0.05$ versus control. **A.** The expression of β1-integrin (ITGB1) and p-FAK in MCF10A cells and BT549 cells; **B.** The levels of β1-integrin (ITGB1) and p-FAK in BT549-sh-Twist and BT549-sh-Ctrl cells; **C.** The β1-integrin (ITGB1), p-AKT, PKM2, LDHA, p53, and G6PD expression in B549 cells transfected with control shRNA (BT549-sh-Ctrl) or β1-integrin shRNA (BT549-sh-ITGB1); **D.** BT549 cells were treated with PF-562271. Expression of p-FAK, p-AKT, PKM2, LDHA, p53, and G6PD was determined.

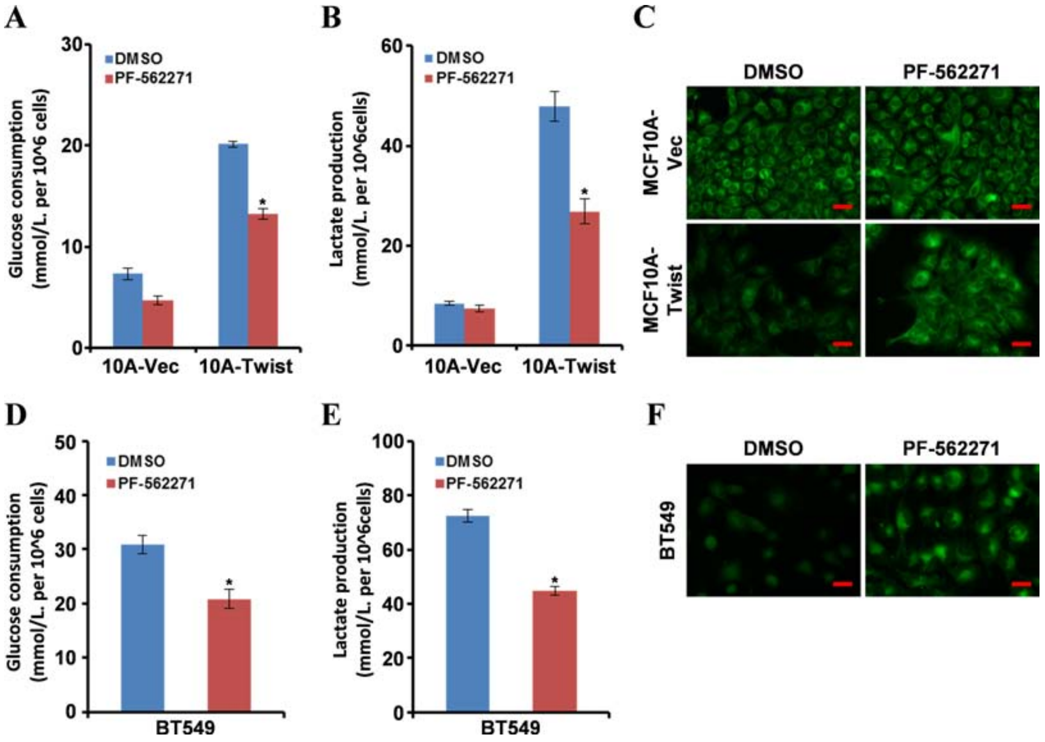

Supplementary Figure S7: The changes in the phenotype of energy metabolism reprogramming in MCF10A-Twist cells or BT549 cells treated with PF-562271. **A, B.** After treated with or without PF-562271, glucose consumption and lactate production of MCF10A-Twist cells were detected. *$P < 0.05$ versus control. **C.** Fluorescence microscope analysis of mitochondrial mass in MCF10A-Twist cells treated with or without PF-562271 by Mito-Tracker Green staining. Magnification, x200. Scale bars, 100 μm. **D, E.** After treated with or without PF-562271, glucose consumption and lactate production of BT549 cells were detected. *$P < 0.05$ versus control. **F.** Fluorescence microscope analysis of mitochondrial mass in BT549 cells treated with or without PF-562271 by Mito-Tracker Green staining. Magnification, x200. Scale bars, 100 μm.

Supplementary Table S1. Quantitative real-time PCR primers

Gene ID	Primer (5′-3′)	Sequence
TWIST	Forward	GGAGTCCGCAGTCTTACGAG
	Reverse	TCTGGAGGACCTGGTAGAGG
G6PD	Forward	TGAGCCAGATAGGCTGGAA
	Reverse	TAACGCAGGCGATGTTGTC
PKM2	Forward	ATGAGTACCATGCGGAGACC
	Reverse	TGTCTAGAGCCACAGCAACG
LDHA	Forward	GCCTGTATGGAGTGGAATGAA
	Reverse	CCAGGATGTGTAGCCTTTGAG
ENO1	Forward	GCCGGCTTTACGTTCACCTC
	Reverse	GTTGAAGCACCACTGGGCAC
PGK1	Forward	ACAATGGAGCCAAGTCGGTAG
	Reverse	GCCTACACAGTCCTTCAAGAAC
TPI1	Forward	GAGCTGATTGGGCAGAAAGTG
	Reverse	CCCAATGCAGGCGATTACTC
p53	Forward	CTGCCCTCAACAAGATGTTTTG
	Reverse	CTATCTGAGCAGCGCTCATGG
β-actin	Forward	CCCTGTGCTGCTCACCGA
	Reverse	ACAGTGTGGGTGACCCCGTC
